# CAVI (Cardio-Ankle Vascular Index) as an independent predictor of hypertensive response to exercise

**DOI:** 10.1186/s12872-024-03807-0

**Published:** 2024-03-19

**Authors:** C. Wuttichaipradit, C. Yodwut, P. Sukhum, K. Hengrussamee, M. Treesong, S. Thiangtham, B. Samut, A. Tunhasiriwet, T. Yingchoncharoen

**Affiliations:** 1 Bangkok Heart Hospital, Bangkok, Thailand; 2 Faculty of Medicine, Ramathibodi Hospital, Mahidol University, Bangkok, Thailand

**Keywords:** Hypertensive response to exercise (HRE), Cardio-ankle vascular index (CAVI), Arterial stiffness, Treadmill stress test

## Abstract

**Objectives:**

Hypertensive response to exercise (HRE) is related to the development of future hypertension, cardiovascular morbidity, and mortality, independent of resting blood pressure. We hypothesized that arterial stiffness as measured by cardio-ankle vascular index (CAVI) could be an independent predictor of HRE.

**Materials and methods:**

Retrospective chart review of patients participated in the preventive health program at the Bangkok Heart Hospital who underwent both CAVI and treadmill stress testing on the same day between June and December 2018 were performed. Variables for the prediction of HRE were analyzed using univariate analysis, and significant variables were entered into multiple logistic regression. An ROC curve was created to test the sensitivity and specificity of CAVI as a predictor of HRE.

**Results:**

A total of 285 participants (55.1% female) were enrolled in this study. There were 58 patients (20.4%) who met the HRE definition (SBP > 210 mmHg in males, SBP > 190 mmHg in females, or DBP > 110 mmHg in both males and females), with a mean age of 46.4 12.8 years. In univariate analysis, age, systolic blood pressure at rest, diastolic blood pressure at rest, pulse pressure at rest, diabetes mellitus, hypertension, dyslipidemia, history of beta-blocker, and CAVI results were statistically significant. Multiple logistic regression revealed that CAVI and systolic blood pressure were statistically significant predictors of HRE with OR of 5.8, 95%CI: 2.9–11.7, *P* < 0.001 and OR 1.07, 95%CI: 1.03–1.10, *P* = 0.001 respectively. ROC curve analysis of the CAVI revealed an AUC of 0.827 (95%CI: 0.76–0.89, *p* < 0.001), and the sensitivity and specificity of cut-point CAVI > 8 were 53% and 92%, respectively.

**Conclusion:**

This study demonstrated that CAVI is an independent predictor of hypertensive response to exercise. Additionally, the findings suggest that CAVI > 8 can be a valuable tool in identifying individuals at risk for hypertensive responses during exercise.

## Introduction

Abnormally exaggerated elevation in systolic blood pressure during exercise is known as the hypertensive response to exercise (HRE). Although there was no universal definition of HRE, the contemporary studies defined HRE as systolic blood pressure exceeding the 90th percentile (approximately a systolic blood pressure of > 210 mmHg in men and > 190 mmHg in women) or a difference between peak and baseline systolic blood pressure of at least 60 mmHg in men and at least 50 mmHg in women during exercise testing [1–3]. Exaggerated increases in blood pressure that occur during exercise have been demonstrated to enhance the risk of developing hypertension [3, 4] and the incidence of left ventricular hypertrophy in normotensive people [5, 6]. Furthermore, several studies have shown that the HRE might serve as a prognostic indicator [2, 7, 8]. Increased peripheral vascular resistance and decreased endothelial function, which lead to poor vasodilation during exercise, are likely the main reasons why exercise raises blood pressure more than usual [9, 10]. The hyperactivity of sympathetic nerves or thickening of the arteriolar wall that affects the ability of the wall to respond to vasoconstrictor stimuli can both be used to explain these inadequate responses of peripheral vascular resistance. As measured by pulse wave velocity (PWV), increased arterial stiffness has also been linked to HRE, but there has not been enough research to prove this linkage.

The cardio-ankle vascular index (CAVI), a novel indicator of arterial stiffness of global arterial segments, was calculated quickly from ankle brachial index (ABI) measurements [11]. Theoretically, CAVI is less dependent on blood pressure than PWV as the blood pressure was corrected in the formula of calculation [12, 13]. CAVI is a predictive indicator in individuals with cerebrovascular illness [14], coronary heart disease [15], hypertension [16], diabetes mellitus [17], chronic renal disease [18], metabolic syndrome [19], and obstructive sleep apnea [20]. In addition, CAVI is recognized as a sensitive method for detecting minor changes in major arteries before a functional impairment is evident [21]. A CAVI score of 8.0 or less is regarded as normal, a score of 9.0 or more indicates probable arteriosclerosis, and a score between 8.0 and 9.0 is regarded as borderline [22]. This study aims to investigate the potential role of CAVI in predicting hypertensive responses to exercise, offering crucial insights into how arterial stiffness relates to blood pressure changes induced by exercise. Understanding this association could have significant implications for the risk stratification and management of patients with HRE.

## Methods

### Study participants

**Fig. 1:**
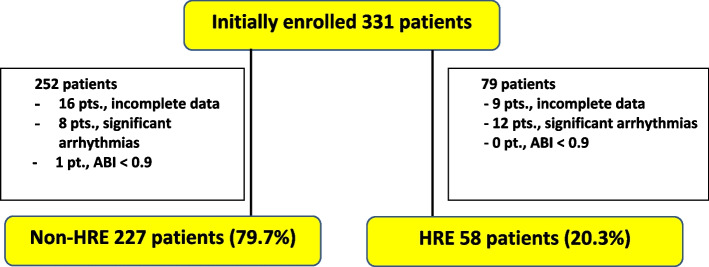
Diagram of recruited participants into the study. *ABI* Ankle brachial index, *HRE* Hypertensive response to exercise

The institutional review board of BDMS health research center, Bangkok Dusit Medical Services, approved the study. All methodologies were performed in accordance with the relevant guidelines and regulations by Ethical approval and waived consent to participants. Patients who participated in the health preventive program at the Bangkok hospital between June and December 2018 and underwent both the CAVI and TST on the same day were initially enrolled in this study. Retrospective chart reviews were conducted in the track care system. The inclusion criteria included age between 18 and 80 years, specifically available data from the treadmill stress test and the CAVI, sinus rhythm with a verified ECG, and the necessary data from the tracking care system is obtained from the complete history checklist with consent approval that the patients must execute prior to the anticipated check-up program. These data consist of general baseline characteristics, past medical illness, and medicine used that might impact the ABI and CAVI results (as shown in Table 1). Patients with significant supraventricular tachyarrhythmias (atrial fibrillation, supraventricular tachycardia, couplet PACs, frequent PACs), or ventricular arrhythmias (sustained ventricular tachycardia, non-sustained ventricular tachycardia, couplet PVCs, frequent PVCs), as documented by an ECG during CAVI measurement or TST, were excluded from the study. In addition, patients with substantial peripheral artery disease, defined as an ABI less than 0.9, were also excluded. For the initial screening phase of this study, 331 participants were enrolled. Finally, we found 285 participants who were eligible for this study protocol (Fig. 1).

**Table 1:** Baseline characteristics in total population

Baseline characteristics	Number (%)
Sex (female)	**157 (55.1%)**
Diabetes Mellitus	**13 (4.6%)**
Hypertension	**45 (15.8%)**
Dyslipidemia	**34 (11.9%)**
Smoking	**32 (11.2%)**
Alcohol consumption	**81 (28.4%)**
History of coronary artery disease	**4 (1.4%)**
History of cerebrovascular disease	**1 (0.4%)**
History of chronic kidney disease	**0**
History of peripheral arterial disease	**0**
Medicine	
Aspirin	**16 (5.6%)**
Metformin	**0**
ACEI/ARB	**11 (3.9%)**
Beta-blocker	**11 (3.9%)**
Calcium channel blocker	**3 (1.1%)**
Statin	**15 (5.3%)**
Age (years)	**46.4 ± 12.8**
Body weight (Kg)	**65.2 ± 14.3**
Height (cm)	**163.6 ± 8.7**
Heart rate	**70.5 ± 10.9**
Systolic blood pressure (mmHg, rest)	**126.9 ± 16.5**
Diastolic blood pressure (mmHg, rest)	**80.3 ± 10.8**
Peak systolic blood pressure (mmHg)	**172.3 ± 27.8**
Peak diastolic blood pressure (mmHg)	**74.0 ± 13.2**
Resting pulse pressure (mmHg)	**43.3 ± 13.4**
Peak pulse pressure (mmHg)	**98.9 ± 25.9**
Maximal functional capacity (METs)	**10.7 ± 2.2**
Different SBP rest-peak (mmHg)	**51.4 ± 20.5**
Different DBP rest-peak (mmHg)	**-4.3 ± 11.8**
Right ABI	**1.05 ± 0.07**
Left ABI	**1.05 ± 0.07**
CAVI	**7.25 ± 0.98**

*SBP* Systolic blood pressure, *DBP* Diastolic blood pressure, *ABI* Ankle brachial index, *CAVI* Cardio-ankle vascular index.

### Treadmill stress test

Before undergoing the exercise stress test, patients are advised to take several preparatory measures. They should refrain from eating for 4–6 h before the test and avoid caffeine for 24 h, which includes beverages such as coffee, tea, and energy drinks. Smoking or the use of any tobacco products is also discouraged. On the day of the test, patients are instructed to temporarily discontinue certain medications, especially beta-blockers and other AV node blocking agents. During the test, all patients underwent symptom-limited treadmill testing with continuous 12-lead ECG monitoring. The standard Bruce protocol was used, which involves a gradual increase in workload by adjusting the speed and incline every three minutes. Blood pressure readings and a 12-lead ECG copy were taken before starting the exercise, after each stage of the exercise (every three minutes), at the peak of the exercise, and then at 1-min intervals throughout the recovery phase. Observations included the patient's symptoms, resting and peak heart rates, blood pressure fluctuations, and any changes in the ECG. The test was terminated if any of the following occurred: debilitating symptoms such as chest pain, breathlessness, or fatigue; significant cardiac arrhythmias; pronounced ST-segment deviation exceeding 0.2 mV accompanied by typical angina; reaching the age-predicted maximum heart rate, calculated as 220 minus the patient's age; or any unusual blood pressure responses. An abnormal blood pressure response is characterized by a decline in blood pressure greater than 10 mmHg despite an augmented workload along with other signs of ischemia. Furthermore, a systolic blood pressure surpassing 250 mmHg or a diastolic blood pressure exceeding 115 mmHg is identified as an exaggerated hypertensive response [23].

### Cardio-Ankle Vascular Index (CAVI)

CAVI was measured using a VaSera CAVI apparatus from Fukuda Denshi Co. Ltd. in Tokyo, Japan. The subject was placed in a supine position with the head in the middle, and bilateral cuffs were placed on the individual's upper arms and ankles. The tests were performed after 10 min of resting in this position. The brachial and ankle arteries' pressures and waveforms, electrography, phonocardiography, and other measurements, were taken. The following formula calculated CAVI: CAVI = a{(2ρ/∆P) × ln(Ps/Pd)PWV2} + b, where Ps is systolic blood pressure, Pd is diastolic blood pressure, PWV is pulse wave velocity, ∆P is Ps– Pd,ρ is blood density, and a and b are constants [24]. Following that, CAVI was automatically calculated. CAVI > 8 was used to define an abnormally increased arterial stiffness. However, when the ABI ratio is less than 0.9, CAVI is unreliable, and hence this group must be eliminated from this study.

### Hypertensive response to exercise

As mentioned above, there is currently no consensus definition of HRE. HRE was classified in this study as SBP ≥ 210 mmHg in men, SBP ≥ 190 mmHg in women, or DBP ≥ 110 mmHg in both men and women [1–3]. We utilized this value to define HRE since previous studies showed a correlation between HRE and future hypertension development and increased left ventricular hypertrophy [5, 6, 8]. Furthermore, HRE has been shown as a significant predictor of major cardiovascular events [2, 7].

### Statistical analysis

All statistical analyses were performed with SPSS 22.0 for Windows (SPSS Inc., Chicago, IL, USA) software package. Categorical variables were presented as frequencies and percentages, and the Fisher's exact test or Pearson chisquare test was used to compare data between the groups. Continuous variables were presented as mean ± standard deviation and were checked for the normality of distribution using the Kolmogorov–Smirnov test. The normally distributed quantitative variables were compared between the two groups using the independent samples T-test; the non-normally distributed ones were compared between the two groups using the Mann–Whitney U test. We performed a univariable logistic regression analysis to determine relationships between various variables and a hypertensive response to exercise. Significant variables from univariate analysis were included into multivariate logistic regression. Additionally, receiver operating characteristic (ROC) analyses were used to determine the area under the curve (AUC) for CAVI > 8 in predicting HRE. The z test was used to compare the AUC. One-way analysis of variance (ANOVA) was used to compare systolic blood pressure differences at rest and peak exercise between the CAVI 3 groups (< 6, 6–8, > 8). A p-value less than 0.05 was considered statistically significant.

## Results

### Baseline characteristics

Baseline characteristics are exhibited in Table 1. Of 285 participants were finally recruited in this study, 58 patients (20.3%) who matched with HRE definition. 157 participants (55%) were females, small population had pre-existing medical conditions (diabetes mellitus 4.6%, hypertension 15.8%, dyslipidemia 11.9%, coronary artery disease 1.4%, cerebrovascular disease 0.4%), and smoking 11.2%. The minority of patients (about 1–6%) take aspirin, statin, beta-blocker, ACEI/ARB, and calcium channel blocker. The hemodynamic parameters were as follows: the systolic blood pressure at rest was 126.9 ± 16.5 mmHg, the maximum systolic blood pressure was 172.3 ± 27.8 mmHg, the resting pulse pressure was 43.3 ± 13.4 mmHg, and the maximum exercise pulse pressure was 98.9 ± 25.9 mmHg; the difference in systolic blood pressure between resting and peak exercise was 51.4 ± 20.5 mmHg, whereas the difference in diastolic blood pressure between resting and peak exercise was -4.3 ± 11.8 mmHg. The left- and right-ABI measurements yield comparable results (1.05 ± 0.07). The CAVI reported values with a maximum and minimum of 10.35 and 4.35, respectively, and a mean ± SD of 7.25 ± 0.98.

### Univariate logistic regression analysis

Comparison of baseline parameters between non-HRE and HRE groups is presented in Table 2. The results of the univariate logistic regression analysis are displayed in Table 3. Age, diabetes mellitus, hypertension, dyslipidemia, history of beta-blockers, as well as systolic, diastolic, and pulse pressures at rest, along with CAVI, all exhibited statistical significance in the univariate logistic regression analysis.

**Table 2:** Comparison baseline parameters between non-HRE and HRE

**Baseline parameters**	**Non-HRE group N 227**	**HRE group N 58**	*p-value*
Female	**120 (52.8%)**	**37 (63.7%)**	0.135
Age	**43.9 ± 11.6**	**56.2 ± 12.4**	** < 0.001**
BW (kg)	**65.4 ± 14.7**	**64.2 ± 12.6**	0.56
Height (cm)	**164.3 ± 8.6**	**160.7 + 8.6**	0.06
BMI	**24.1 ± 4.1**	**24.7 ± 3.5**	0.25
Resting HR (bpm)	**70.4 ± 11.0**	**70.8 ± 10.8**	0.79
Resting SBP (mmHg)	**117.2 ± 14.4**	**138.6 ± 18.1**	** < 0.001**
Resting DBP (mmHg)	**76.7 ± 10.6**	**84.4 ± 11.4**	** < 0.001**
Resting pulse pressure(mmHg)	**40.4 ± 11.0**	**54.3 ± 16.0**	** < 0.001**
Peak pulse pressure (mmHg)	**92.6 ± 22.3**	**123.6 ± 24.7**	** < 0.001**
Maximal functional capacity (METs)	**11.1 ± 2.02**	**9.40 ± 2.33**	** < 0.001**
Right ABI	**1.04 ± 0.07**	**1.07 ± 0.07**	**0.287**
Left ABI	**1.05 ± 0.07**	**1.06 ± 0.06**	**0.165**
CAVI	**7.0 ± 0.76**	**8.3 ± 1.06**	** < 0.001**
DM	5 (2%)	8 (13.7%)	0.001
HT	24 (10.6%)	21 (36.2%)	< 0.001
Dyslipidemia	19 (8.4%)	15 (25.8%)	0.001
Smoking	28 (12.3%)	4 (6.9%)	0.24
Family history of CAD &CVA	28 (12.3%)	5 (8.6%)	0.43
History of CAD	3 (1.3%)	1 (1.7%)	0.81
History on ASA	8 (3.5%)	2 (3.4%)	0.5
History on statin	9 (3.9%)	6 (10.3%)	0.052
History of ACEIs/ARBs	7 (3%)	4 (6.9%)	0.18
History on beta-blocker	5 (2.2%)	6 (10.3%)	0.004
History on CCB	2 (1%)	1 (1.7%)	0.57

*HRE* Hypertensive response to exercise, *BMI* Body mass index, *SBP* Systolic blood pressure, *DBP* Diastolic blood pressure, *METs* Metabolic equivalent, *ABI* Ankle brachial index, *CAVI* Cardio-ankle vascular index, *DM* Diabetes mellitus, *HT* Hypertension, *CAD* Coronary artery disease, *CVA* Cerebrovascular accident, *ASA* Aspirin, *ACEIs* Angiotensin-converting enzyme inhibitors, *ARB* ARBs: angiotensin receptor blockers, *CCB* Calcium channel blocker.

**Table 3:** Univariate analysis of predictors of HRE

Variables	HRE OR (95% CI)	*p-value*
Age (years)	1.08 (1.06–1.12)	< 0.001
Resting SBP	1.08 (1.06–1.11)	< 0.001
Resting DBP	1.06 (1.04–1.10)	< 0.001
Resting PP	1.08 (1.05–1.11)	< 0.001
CAVI	5.83 (3.60–9.43)	< 0.001
DM	7.1 (2.23–22.63)	0.001
HT	4.8 (2.42–9.49)	< 0.001
DLP	3.82 (1.80–8.10)	< 0.001
Beta-blocker used	5.1 (1.51–17.4)	0.004

*SBP* Systolic blood pressure, *DBP* Diastolic blood pressure, *PP* Pulse pressure, *CAVI* Cardio-ankle vascular index, *DM* Diabetes mellitus, *HT* Hypertension, *DLP* Dyslipidemia, *CI* Confidential interval.

### Multivariate logistic regression analysis

The results are presented in Table 4. Multivariate logistic regression analysis was conducted using two models. Model A included variables such as age, diabetes mellitus, hypertension, dyslipidemia, beta-blocker therapy, and CAVI. However, the results indicated that only CAVI was statistically significant in predicting HRE, with an odds ratio (OR) of 5.8 (95% CI 3.1–10.8, *p* < 0.001). In Model B, which incorporated factors like age, diabetes mellitus, hypertension, systolic blood pressure at rest, diastolic blood pressure at rest, and CAVI, the analysis revealed that both CAVI and systolic blood pressure at rest were statistically significant predictors of HRE, with ORs of 5.8 (95% CI 2.9–11.7, *p* < 0.001) and 1.07 (95% CI 1.03–1.10, *p* = 0.001), respectively.

**Table 4:** Multivariate predictors after adjustment of confounders

Variables	HRE OR (95% CI)	*p-value*
**Model A**		
Age (years)	0.99 (0.96–1.04)	0.85
Diabetes Mellitus	2.12 (0.41–10.9)	0.36
Hypertension	0.48 (0.17–1.32)	0.16
Dyslipidemia	0.72 (0.25–2.04)	0.54
Beta-blocker treatment	0.82 (0.15–4.5)	0.82
**CAVI**	**5.8 (3.1–10.8)**	** < 0.001**
**Model B**		
Age (years)	0.98 (0.94–1.03)	0.56
Diabetes Mellitus	2.1 (0.35–11.9)	0.42
Hypertension	0.85 (0.3–2.3)	0.75
**Resting SBP**	**1.07 (1.03–1.10)**	**0.001**
Resting DBP	1.01 (0.96–1.06)	0.64
**CAVI**	**5.8 (2.9–11.7)**	** < 0.001**

In the multivariate analysis of predictors of hypertensive response to exercise (HRE), Model A and B were created to fit the number of events (58 patients who have HRE). *SBP* Systolic blood pressure, *DBP*: Diastolic blood pressure

### Comparison of the CAVI between HRE and non-HRE

**Fig. 2:**
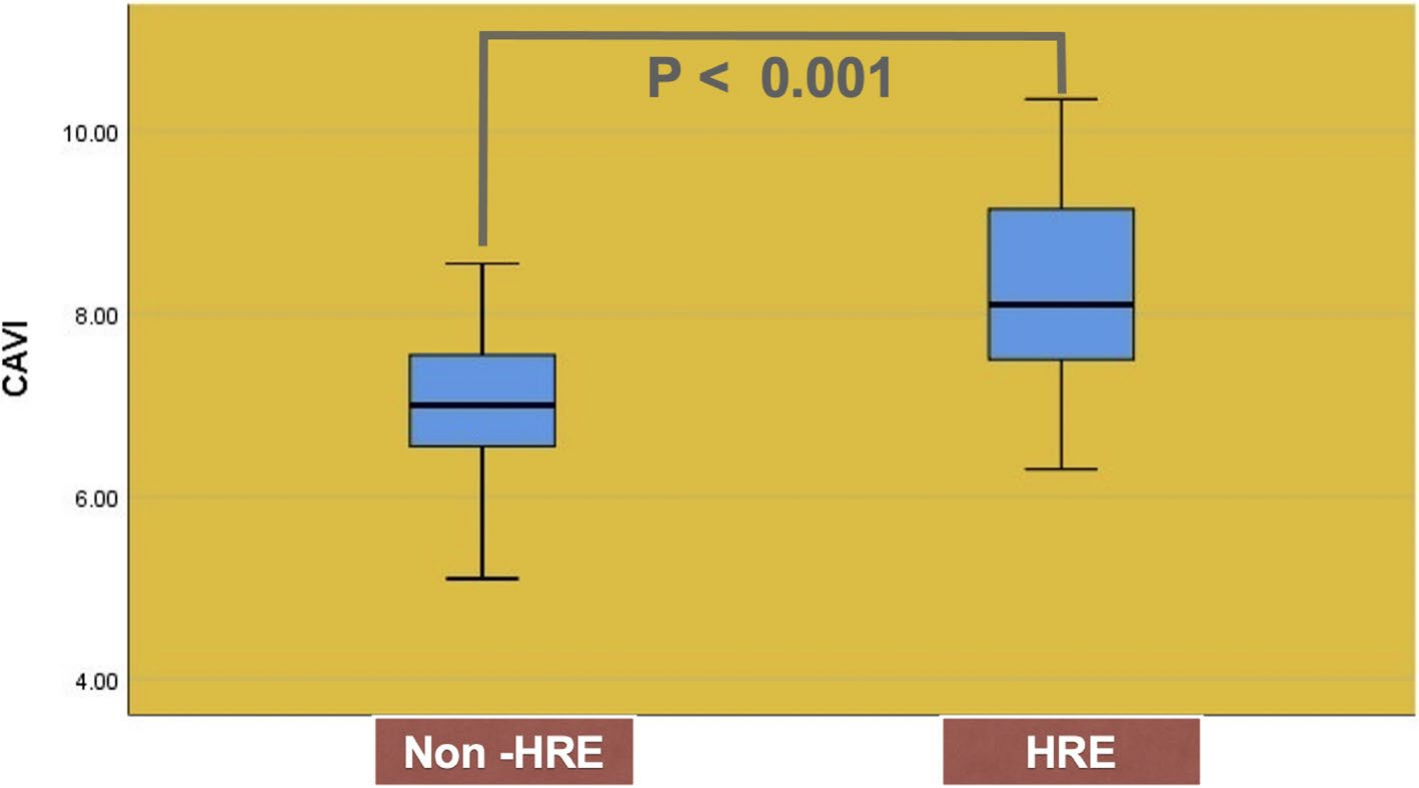
Comparison of the CAVI between non-HRE and HRE group. *HRE* Hypertensive response to exercise, *CAVI* Cardio-ankle vascular index

In the non-HRE group, the CAVI result was 6.9 ± 0.7, whereas in the HRE group, it was 8.3 ± 1.1. A comparison of CAVI between the non-HRE and HRE groups revealed a statistically significant difference, with *p* < 0.001 (see Fig. 2).

### The ROC curve of CAVI to predict HRE

**Fig. 3:**
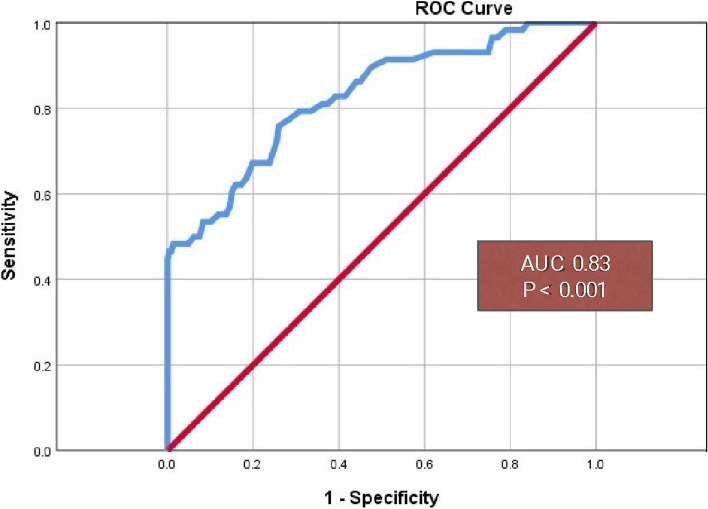
The ROC curve of CAVI to predict HRE. *ROC* Receiver operating characteristic, *AUC* Area under the curve, *CAVI* Cardio-ankle vascular index

The previous study identified an abnormal result when CAVI was greater than 8 [22]. In our study, we adopted a CAVI > 8 as a threshold to predict HRE. ROC curve analysis showed that CAVI was a statistically significant predictor of HRE, with an area under the curve (AUC) of 0.827 (95% CI: 0.76–0.89, *p* < 0.001). Sensitivity and specificity were found to be 53% and 92%, respectively (see Fig. 3).

### Comparison of the different SBP (at rest & peak) between 3 CAVI-groups

**Fig. 4:**
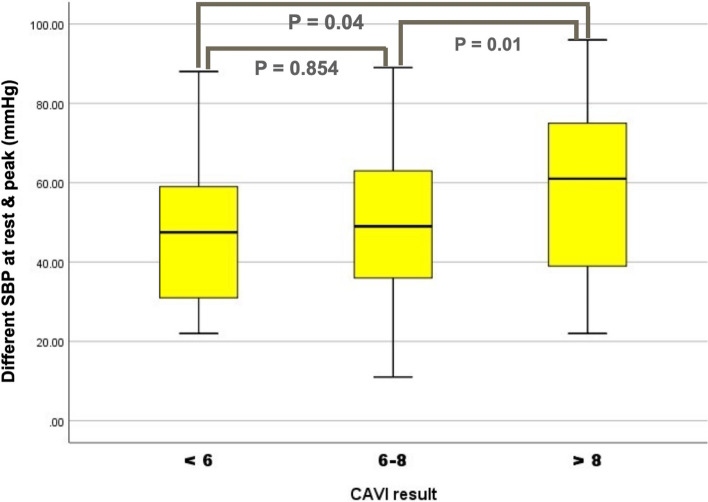
SBP denoted systolic blood pressure. Comparison of the different SBP at rest and peak exercise between 3 groups of CAVI (less than 6, 6–8, and > 8). *SBP* Systolic blood pressure, *DBP* Diastolic blood pressure, *CAVI* Cardio-ankle vascular index

It was examined using the Kolmogorov–Smirnov test, revealing the normality distribution. As a result, in Fig. 4, the CAVI were classified into 3 groups (< 6, 6–8, > 8). There was no statistically significant difference in systolic blood pressure between the CAVI < 6 and the CAVI 6–8 (differences at rest and peak exertion). However, comparing the CAVI 6 and the CAVI > 8, as well as the comparison between the CAVI 6–8 and the CAVI > 8, the result showed a statistically significant difference, *p*-value = 0.04 and 0.01, respectively.

#### Comparison of the pulse pressure in each stage between CAVI <  = 8 and CAVI > 8

**Fig. 5:**
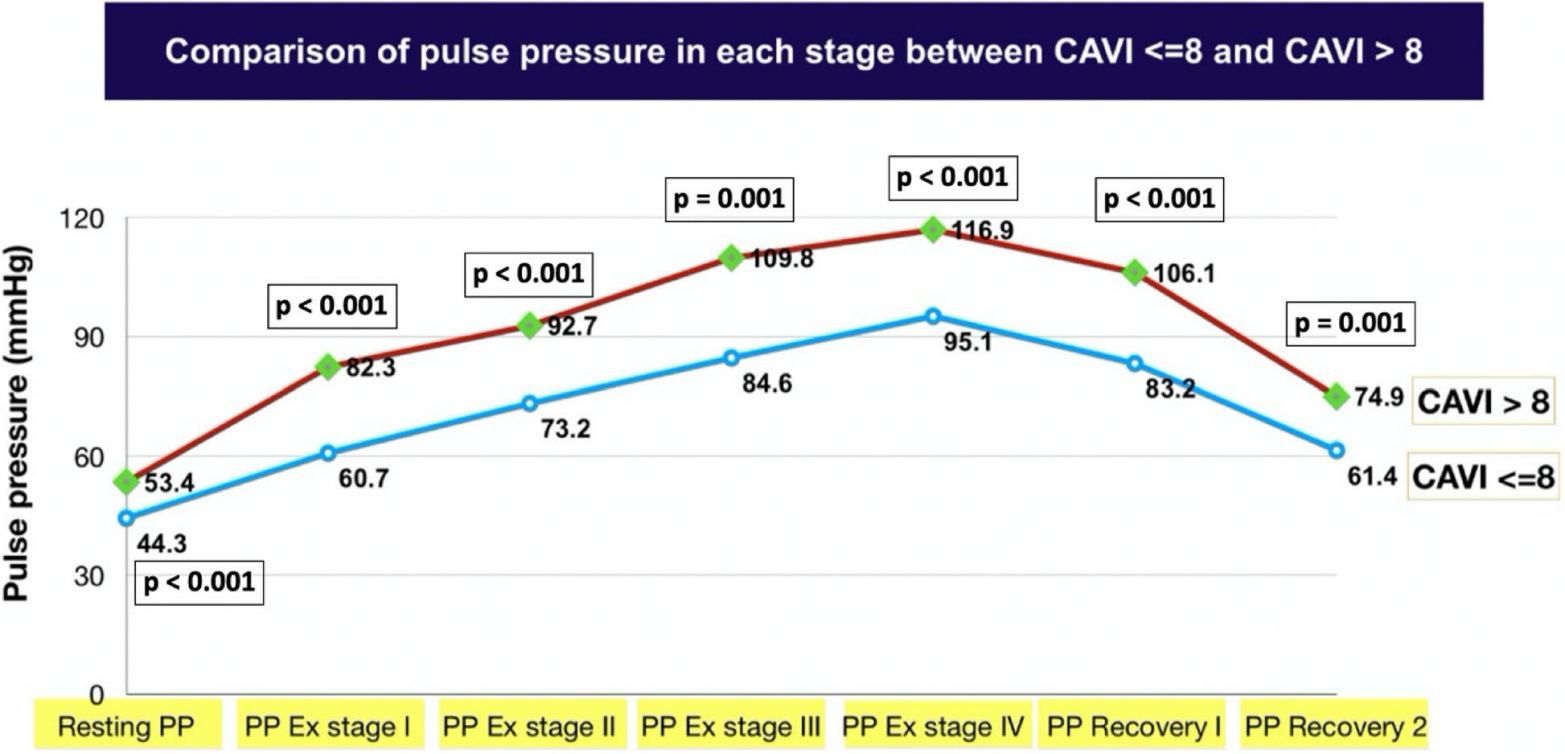
Comparison of the pulse pressure in each stage between CAVI <  = 8 and CAVI > 8: CAVI—cardio-ankle vascular index, *PP* Pulse pressure, *Ex* Exercise, *CAVI* Cardio-ankle vascular index

The findings are shown in Fig. 5. Patients with CAVI > 8 had statistically significant greater pulse pressure at all levels of exercise than patients with CAVI <  = 8. (Include baseline, exercise at every stage, and recovery period).

## Discussion

The current study has demonstrated that arterial stiffness, as measured by CAVI (OR 5.5, 95% CI: 2.8–10.7, *p* < 0.001), and resting pulse pressure (OR 1.05, 95% CI: 1.02–1.08, *p* < 0.001), constitutes statistically significant predictors of HRE. Furthermore, the results of this study have provided new insights into the impact of arterial stiffness on excessive exercise systolic blood pressure. In the non-HRE group, the CAVI result was 6.9 ± 0.7, whereas in the HRE group, it was 8.3 ± 1.1 (*p* < 0.001). Thus, our results substantiate the hypothesis that HRE is mechanistically associated with arterial stiffness, independent of other established cardiovascular disease risk factors.

We have also demonstrated that CAVI, serving as a surrogate marker of arterial stiffness, can discriminate against arterial compliance. This study has identified a CAVI value greater than 8 as the optimal cutoff for predicting HRE. ROC curve analysis has shown that CAVI is a statistically significant predictor of HRE, with an AUC of 0.827 (95% CI: 0.76–0.89, *p* < 0.001), and sensitivity and specificity values of 53% and 92%, respectively.

These findings suggest that CAVI could have clinical utility in identifying arterial stiffness in individuals at an elevated risk of cardiovascular disease. However, it's important to note that our findings may not be generalizable to patients with established cardiovascular disease, as our study population consisted of individuals at low and intermediate ASCVD risk.

### CAVI and resting pulse pressure as independent predictors of hypertensive response to exercise

When considering hypertensive response to exercise (HRE) as the dependent variable, the significant univariate predictors for HRE included age, DM, HT, dyslipidemia, beta-blocker history, exercise time, resting pulse pressure, and CAVI. Interestingly, conventional ASCVD risk factors were not significant in the multivariate analysis.

### Cutoff CAVI

To the best of our knowledge, this study marks the first attempt to propose a CAVI cutoff value of 8.0 for the early detection of hypertensive responses to exercise. This value is recommended as the optimal threshold for screening arterial stiffness in asymptomatic populations, based on previously published research [25]. In 2007–2008, the largest longitudinal cohort study in Thailand was conducted on 3,807 Electricity Generating Authority of Thailand (EGAT) employees. The ideal CAVI threshold for coronary artery disease (CAD) is 8. Incorporating CAVI into the conventional risk score (RAMA-EGAT) enhances the C-statistics from 0.72 to 0.85 and leads to a 27% net reclassification improvement (NRI) (*p*< 0.0001) [15]. Additionally, arterial stiffness, as measured by CAVI in this population, may enhance the predictive capacity for future major adverse cardiovascular events (MACEs). Individuals with CAVI > 9 had a 1.34-fold increased risk of MACEs (95 percent CI: 1.01, 1.79) compared to those with CAVI < 9 [26].

### SBP and DBP Cut-off for HRE

This study utilized a cut-off value of SBP ≥ 210 mmHg for men and ≥ 190 mmHg for women, or DBP ≥ 110 mmHg for both genders. These thresholds were established based on the exceeding of the 90th centile blood pressure responses to maximal or peak intensity exercise. This cut-off has been demonstrated to correlate with future hypertension [5, 6], increased left ventricular hypertrophy [8], and significant predictors of major adverse cardiac events [2, 7]. Sharman et al. [27] have similarly shown that hypertensive response to exercise (HRE), using the same cut-point as our study, can aid in the detection of masked hypertension, as identified through 24-h ambulatory blood pressure monitoring (ABPM). Furthermore, CAVI has also been identified as an independent risk factor for masked uncontrolled hypertension [28], with the CAVI results in the masked hypertension group in one study ranging from approximately 8.2 to 9.9, a range that closely aligns with the CAVI values observed in the HRE group in our study. Endothelial dysfunction and increased arterial stiffness have been proposed as the mechanisms underlying HRE [29]. Chung et al. [30] have demonstrated that arterial stiffness, assessed using brachial-ankle pulse wave velocity (baPWV), serves as an independent predictor of HRE, consistent with the same cut-off point as in our study. Moreover, this study also found a higher prevalence of HRE in women (56%), which is consistent with our study's findings, where 67% of women exhibited HRE. Hence, this cut-off value represents an appropriate threshold for HRE that can be associated with the CAVI results observed in our study.

### Validated CAVI by pulse wave velocity

In a cohort nationwide registry in Japan, the CAVI reference value was investigated by measuring CAVI in 4,542 patients with at least one cardiovascular risk factor and baPWV in 1,737 of these 4,542 patients on the same day. A significant and positive correlation was observed between CAVI and baPWV (*r* = 0.50, *p*0.001). CAVI was 8.303 for baPWV at 14 m/s and 9.059 for baPWV at 18 m/s as calculated by the regression line [31].

### Clinical implications

This study is the first to demonstrate a significant correlation between arterial stiffness using CAVI and hypertensive response during exercise (HRE). Previous study has shown the linkage between arterial stiffness using pulse wave velocity (PWV) and HRE [32]. Although both methods of measurement are practicable, the marker of arterial stiffness using CAVI has an advantage beyond the PWV (carotid-femoral) since it is less affected by blood pressure at the time of measurement. The blood pressure (BP) response to exercise is a significant predictor of cardiovascular disease and prognosis. The results of this study have the following consequence for clinical practice: As is well known, several factors, such as technical measurements, the patient clenching his arm during exercise, and sleep deprivation, can affect how the blood pressure responds to exercise. CAVI can assist in separating an HRE from a false positive when we discover a patient who has a hypertensive response during exercise [33]. The blood pressure response to exercise is a significant predictor of cardiovascular disease and prognosis. Thus, regular monitoring of blood pressure is crucial for patients with HRE and CAVI > 8 to ensure early detection of any potential cardiovascular complications. This comprehensive approach helps in accurately diagnosing and managing patients with exercise-induced hypertension. Additionally, healthcare professionals should provide comprehensive education and guidance to these patients on lifestyle modifications and medication adherence to effectively manage their condition.

### Study limitations

Our study has certain limitations. Firstly, it was a retrospective, single-center study that relied on patient medical records from a health promotion center, primarily involving participants with low to moderate risk. There was an inadequate representation of individuals with heart disease or those at high risk for it. Secondly, CAVI is a relatively new measurement with notable interobserver and interobservers variability. Given that multiple technicians conducted our CAVI studies, there may have been variations in measurement techniques and variations in the emotional stress conditions experienced by the patients.

### Future direction

Hypertensive response to exercise is linked to endothelial dysfunction, decreased proximal aortic compliance, and increased neurohormonal activation, which may explain why cardiovascular disease will happen in the future [10, 34]. Future research needs to find out if a hypertensive response to exercise is linked to heart disease in the future. Moreover, a delayed blood pressure recovery ratio may indicate increased arterial stiffness in hypertensive patients with reduced aerobic exercise capacity [35]. Furthermore, research on this correlation and future cardiovascular disease is required Link to future HT or ASCVD.

An exaggerated blood pressure response to exercise emerged as a significant and independent risk marker for the development of hypertension from a high-normal state. Therefore, exercise testing can offer valuable insights in identifying individuals at a higher likelihood of developing future hypertension, warranting focused preventive interventions. This finding underscores its potential utility in early risk assessment for both hypertension and ASCVD (atherosclerotic cardiovascular disease) [8].

## Conclusion

This study has demonstrated that CAVI is an independent predictor of hypertensive responses to exercise. Additionally, the findings suggest that a CAVI greater than 8 can serve as a valuable tool for identifying individuals at risk of hypertensive responses during exercise. Furthermore, the incorporation of CAVI measurements into routine exercise assessments could potentially aid in the early detection and intervention for those at risk of developing exercise-induced hypertension. Further evaluation may be necessary for a comprehensive assessment.

## Data Availability

For the raw data sharing, please request to the corresponding author of this article.

## Abbreviations

HRE: Hypertensive response to exercise
CAVI: Cardio-ankle vascular index
SBP: Systolic blood pressure
DBP: Diastolic blood pressure
PP: Pulse pressure
PWV: Pulse wave velocity
ABI: Ankle brachial index
TST: Treadmill stress test
PP: Pulse pressure
ACEIs: Angiotensin-converting enzyme inhibitors
ARBs: Angiotensin receptor blockers
CCB: Calcium channel blocker
ROC: Receiver operating characteristic
AUC: Area under the curve
ANOVA: Analysis of variance
Ex: Exercise
ABPM: Ambulatory blood pressure monitoring
